# 3-[(*E*)-2-Phenyl­ethen­yl]-1*H*-indole-6-carbonitrile

**DOI:** 10.1107/S1600536811054225

**Published:** 2011-12-21

**Authors:** Yu-Hua Ge, Dong-En Wu, Yang-Hui Luo

**Affiliations:** a Ordered Matter Science Research Center, College of Chemistry and Chemical Engineering, Southeast University, Nanjing 210096, People’s Republic of China

## Abstract

In the title compound, C_17_H_12_N_2_, the inter­planar angle between the indole mean plane [max.deviation 0.030 (1) Å] and the phenyl ring is 24.32 (7)°. In the crystal, inter­molecular N—H⋯N C hydrogen bonds form zigzag chains in the *a*-axis direction augmented by weak C—H⋯N C contacts.

## Related literature

For indole derivatives as drug inter­mediates, see: Kunzer & Wendt (2011 ▶).
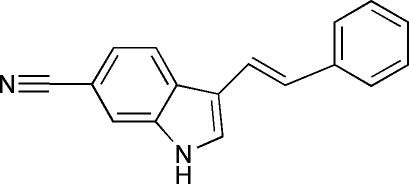


## Experimental

### 

#### Crystal data

C_17_H_12_N_2_
*M* *_r_* = 244.29
Orthorhombic, 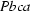

*a* = 9.689 (8) Å
*b* = 7.440 (6) Å
*c* = 35.53 (3) Å
*V* = 2561 (4) Å^3^
*Z* = 8
Mo *K*α radiation
μ = 0.08 mm^−1^
*T* = 293 K
0.20 × 0.20 × 0.20 mm

#### Data collection

Rigaku SCXmini diffractometer
Absorption correction: multi-scan (*CrystalClear*; Rigaku, 2005 ▶) *T*_min_ = 0.985, *T*_max_ = 0.985
16536 measured reflections
2263 independent reflections
1867 reflections with *I* > 2σ(*I*)
*R*_int_ = 0.027

#### Refinement

*R*[*F* ^2^ > 2σ(*F* ^2^)] = 0.038
*wR*(*F* ^2^) = 0.127
*S* = 1.16
2263 reflections
172 parameters
H-atom parameters constrained
Δρ_max_ = 0.13 e Å^−3^
Δρ_min_ = −0.20 e Å^−3^

### 

Data collection: *CrystalClear* (Rigaku, 2005 ▶); cell refinement: *CrystalClear*; data reduction: *CrystalClear*; program(s) used to solve structure: *SHELXS97* (Sheldrick, 2008 ▶); program(s) used to refine structure: *SHELXL97* (Sheldrick, 2008 ▶); molecular graphics: *DIAMOND* (Brandenburg & Putz, 2005 ▶); software used to prepare material for publication: *SHELXL97*.

## Supplementary Material

Crystal structure: contains datablock(s) I, global. DOI: 10.1107/S1600536811054225/gg2065sup1.cif

Structure factors: contains datablock(s) I. DOI: 10.1107/S1600536811054225/gg2065Isup2.hkl

Supplementary material file. DOI: 10.1107/S1600536811054225/gg2065Isup3.cml

Additional supplementary materials:  crystallographic information; 3D view; checkCIF report

## Figures and Tables

**Table 1:** Hydrogen-bond geometry (Å, °)

*D*—H⋯*A*	*D*—H	H⋯*A*	*D*⋯*A*	*D*—H⋯*A*
N1—H1*A*⋯N2^i^	0.90	2.19	3.043 (3)	158
C5—H5*A*⋯N2^ii^	0.93	2.66	3.416 (4)	138

Symmetry codes: (i) 
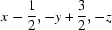
; (ii) 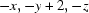.

**Fig. 1.**
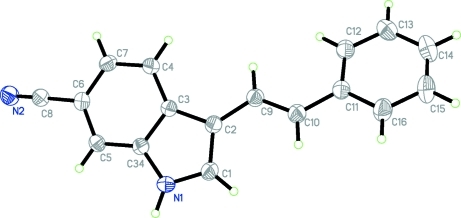
The molecular structure of the title compound with the atom-labelling scheme. Displacement ellipsoids are drawn at the 30% probability level. H atoms are represented as small spheres of arbitrary radii.

**Fig. 2.**
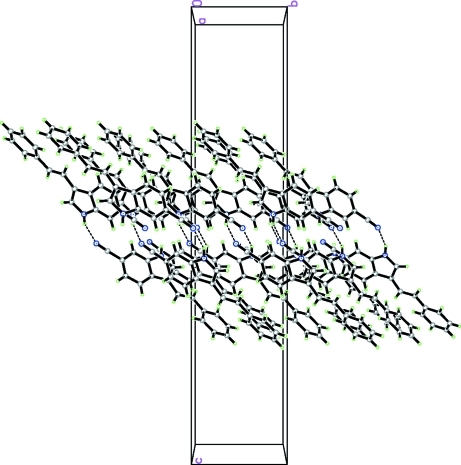
A packing view down the a axis showing the three dimensionnal network. Intermolecular hydrogen bonds are shown as dashed lines.

## supplementary crystallographic information

### Comment

Derivatives of indole are important chemical materials because they are excellent drug intermediates for many pharmaceutical products (Kunzer, *et al.*,2011). As part of our interest in these materials, we report here the crystal structure of the title compound C_17_H_12_N_2_.

The molecular structure of the title compound is shown in Fig. 1. a dihedral angle of 24.32 (7)° between the planes of the indole and benzene rings is observed.

In the crystal, there are intermolecular N—H···O hydrogen bonds and no significant intermolecular π–π interactions [minimum ring centroid separation, 7.440 (5) Å]. (Fig. 2).

### Experimental

The title compound *E*-3-phenyl vinyl-6-cynaindole was obtained economically, Crystals of of it suitable for X-ray diffraction were obstained by slow evaporation of a ethanol solution.

### Refinement

All H atoms attached to C atoms and O atoms were fixed geometrically and treated as riding with C—H = 0.93 Å (CH) and N—H = 0.86 Å with *U*_iso_(H) = 1.2*U*_eq_(C and N).

### Crystal data

**Table:**

C_17_H_12_N_2_	*F*(000) = 1024
*M**_r_* = 244.29	*D*_x_ = 1.267 Mg m^−^^3^
Orthorhombic, *P**b**c**a*	Mo *K*α radiation, λ = 0.71073 Å
Hall symbol: -P 2ac 2ab	Cell parameters from 2263 reflections
*a* = 9.689 (8) Å	θ = 1.2–25.0°
*b* = 7.440 (6) Å	µ = 0.08 mm^−^^1^
*c* = 35.53 (3) Å	*T* = 293 K
*V* = 2561 (4) Å^3^	Prism, blue
*Z* = 8	0.20 × 0.20 × 0.20 mm

### Data collection

**Table:**

Rigaku SCXmini diffractometer	2263 independent reflections
Radiation source: fine-focus sealed tube	1867 reflections with *I* > 2σ(*I*)
graphite	*R*_int_ = 0.027
Detector resolution: 13.6612 pixels mm^-1^	θ_max_ = 25.0°, θ_min_ = 1.2°
CCD_Profile_fitting scans	*h* = −11→11
Absorption correction: multi-scan (*CrystalClear*; Rigaku, 2005)	*k* = −7→8
*T*_min_ = 0.985, *T*_max_ = 0.985	*l* = −42→42
16536 measured reflections	

### Refinement

**Table:**

Refinement on *F*^2^	Primary atom site location: structure-invariant direct methods
Least-squares matrix: full	Secondary atom site location: difference Fourier map
*R*[*F*^2^ > 2σ(*F*^2^)] = 0.038	Hydrogen site location: inferred from neighbouring sites
*wR*(*F*^2^) = 0.127	H-atom parameters constrained
*S* = 1.16	*w* = 1/[σ^2^(*F*_o_^2^) + (0.069*P*)^2^ + 0.3176*P*] where *P* = (*F*_o_^2^ + 2*F*_c_^2^)/3
2263 reflections	(Δ/σ)_max_ < 0.001
172 parameters	Δρ_max_ = 0.13 e Å^−^^3^
0 restraints	Δρ_min_ = −0.20 e Å^−^^3^

### Special details

**Table:**

Geometry. All e.s.d.'s (except the e.s.d. in the dihedral angle between two l.s. planes) are estimated using the full covariance matrix. The cell e.s.d.'s are taken into account individually in the estimation of e.s.d.'s in distances, angles and torsion angles; correlations between e.s.d.'s in cell parameters are only used when they are defined by crystal symmetry. An approximate (isotropic) treatment of cell e.s.d.'s is used for estimating e.s.d.'s involving l.s. planes.
Refinement. Refinement of *F*^2^ against ALL reflections. The weighted *R*-factor *wR* and goodness of fit *S* are based on *F*^2^, conventional *R*-factors *R* are based on *F*, with *F* set to zero for negative *F*^2^. The threshold expression of *F*^2^ > σ(*F*^2^) is used only for calculating *R*-factors(gt) *etc*. and is not relevant to the choice of reflections for refinement. *R*-factors based on *F*^2^ are statistically about twice as large as those based on *F*, and *R*- factors based on ALL data will be even larger.

### Fractional atomic coordinates and isotropic or equivalent isotropic displacement parameters (Å^2^)

**Table:**

	*x*	*y*	*z*	*U*_iso_*/*U*_eq_	
N1	−0.14124 (13)	0.36176 (18)	0.04780 (4)	0.0497 (4)	
H1A	−0.1933	0.3603	0.0268	0.060*	
N2	0.17232 (16)	1.0338 (2)	0.01757 (4)	0.0620 (4)	
C1	−0.13711 (17)	0.2312 (2)	0.07463 (5)	0.0515 (4)	
H1B	−0.1876	0.1250	0.0738	0.062*	
C2	−0.04867 (15)	0.2774 (2)	0.10301 (4)	0.0437 (4)	
C3	0.00642 (14)	0.4503 (2)	0.09281 (4)	0.0388 (4)	
C4	0.09969 (16)	0.5690 (2)	0.10937 (4)	0.0448 (4)	
H4A	0.1368	0.5438	0.1329	0.054*	
C5	−0.01787 (14)	0.6535 (2)	0.03886 (4)	0.0426 (4)	
H5A	−0.0577	0.6824	0.0158	0.051*	
C6	0.07849 (15)	0.7645 (2)	0.05558 (4)	0.0435 (4)	
C7	0.13635 (17)	0.7231 (2)	0.09078 (4)	0.0477 (4)	
H7A	0.2001	0.8010	0.1016	0.057*	
C8	0.12868 (16)	0.9173 (2)	0.03509 (4)	0.0486 (4)	
C9	−0.00992 (16)	0.1678 (2)	0.13506 (4)	0.0470 (4)	
H9A	0.0605	0.2106	0.1504	0.056*	
C10	−0.06676 (17)	0.0115 (2)	0.14427 (5)	0.0508 (4)	
H10A	−0.1435	−0.0224	0.1302	0.061*	
C11	−0.02405 (17)	−0.1135 (2)	0.17358 (4)	0.0469 (4)	
C12	0.10257 (18)	−0.1029 (2)	0.19183 (5)	0.0534 (4)	
H12A	0.1627	−0.0094	0.1861	0.064*	
C13	0.1402 (2)	−0.2282 (3)	0.21825 (5)	0.0649 (5)	
H13A	0.2253	−0.2186	0.2302	0.078*	
C14	0.0534 (2)	−0.3680 (3)	0.22722 (6)	0.0756 (6)	
H14A	0.0794	−0.4527	0.2451	0.091*	
C15	−0.0719 (2)	−0.3811 (3)	0.20954 (6)	0.0795 (7)	
H15A	−0.1315	−0.4749	0.2155	0.095*	
C16	−0.1100 (2)	−0.2561 (3)	0.18295 (5)	0.0639 (5)	
H16A	−0.1949	−0.2673	0.1710	0.077*	
C34	−0.05265 (14)	0.4975 (2)	0.05786 (4)	0.0403 (4)	

### Atomic displacement parameters (Å^2^)

**Table:**

	*U*^11^	*U*^22^	*U*^33^	*U*^12^	*U*^13^	*U*^23^
N1	0.0468 (7)	0.0499 (8)	0.0525 (8)	−0.0053 (6)	−0.0124 (6)	0.0016 (6)
N2	0.0596 (9)	0.0647 (10)	0.0618 (9)	−0.0127 (8)	−0.0015 (7)	0.0148 (8)
C1	0.0481 (9)	0.0448 (10)	0.0616 (10)	−0.0067 (7)	−0.0061 (8)	0.0041 (8)
C2	0.0403 (8)	0.0420 (9)	0.0487 (9)	0.0003 (7)	−0.0001 (7)	0.0007 (7)
C3	0.0354 (7)	0.0397 (8)	0.0412 (8)	0.0043 (6)	0.0014 (6)	−0.0011 (6)
C4	0.0484 (9)	0.0457 (9)	0.0402 (8)	0.0001 (7)	−0.0032 (7)	−0.0013 (7)
C5	0.0398 (8)	0.0457 (9)	0.0423 (8)	0.0066 (7)	0.0012 (6)	0.0023 (7)
C6	0.0417 (8)	0.0417 (9)	0.0472 (8)	0.0023 (7)	0.0076 (7)	0.0019 (7)
C7	0.0490 (9)	0.0466 (9)	0.0476 (9)	−0.0072 (7)	−0.0015 (7)	−0.0044 (7)
C8	0.0459 (9)	0.0509 (10)	0.0491 (9)	−0.0018 (8)	0.0015 (7)	0.0021 (8)
C9	0.0457 (8)	0.0454 (9)	0.0498 (9)	−0.0013 (7)	−0.0007 (7)	0.0016 (7)
C10	0.0488 (9)	0.0495 (10)	0.0540 (9)	−0.0028 (8)	−0.0021 (7)	0.0032 (8)
C11	0.0532 (9)	0.0432 (9)	0.0443 (9)	0.0014 (7)	0.0061 (7)	−0.0008 (7)
C12	0.0602 (10)	0.0498 (10)	0.0503 (9)	0.0006 (8)	0.0041 (8)	−0.0005 (8)
C13	0.0706 (12)	0.0697 (13)	0.0543 (10)	0.0150 (10)	−0.0015 (9)	0.0040 (9)
C14	0.0897 (15)	0.0720 (15)	0.0653 (12)	0.0171 (12)	0.0135 (11)	0.0233 (10)
C15	0.0866 (15)	0.0673 (14)	0.0845 (15)	−0.0052 (11)	0.0183 (12)	0.0279 (12)
C16	0.0609 (11)	0.0588 (12)	0.0722 (12)	−0.0072 (9)	0.0046 (9)	0.0116 (10)
C34	0.0347 (7)	0.0417 (8)	0.0446 (8)	0.0037 (6)	0.0009 (6)	−0.0016 (7)

### Geometric parameters (Å, °)

**Table:**

N1—C1	1.361 (2)	C7—H7A	0.9300
N1—C34	1.372 (2)	C9—C10	1.328 (2)
N1—H1A	0.8999	C9—H9A	0.9300
N2—C8	1.148 (2)	C10—C11	1.457 (2)
C1—C2	1.367 (2)	C10—H10A	0.9300
C1—H1B	0.9300	C11—C12	1.390 (3)
C2—C3	1.439 (2)	C11—C16	1.389 (3)
C2—C9	1.450 (2)	C12—C13	1.373 (3)
C3—C34	1.412 (2)	C12—H12A	0.9300
C3—C4	1.394 (2)	C13—C14	1.375 (3)
C4—C7	1.370 (2)	C13—H13A	0.9300
C4—H4A	0.9300	C14—C15	1.370 (3)
C5—C34	1.385 (2)	C14—H14A	0.9300
C5—C6	1.380 (2)	C15—C16	1.376 (3)
C5—H5A	0.9300	C15—H15A	0.9300
C6—C7	1.405 (2)	C16—H16A	0.9300
C6—C8	1.435 (2)		
			
C1—N1—C34	108.91 (14)	C10—C9—H9A	117.3
C1—N1—H1A	126.0	C2—C9—H9A	117.3
C34—N1—H1A	125.1	C9—C10—C11	128.16 (16)
N1—C1—C2	110.82 (15)	C9—C10—H10A	115.9
N1—C1—H1B	124.6	C11—C10—H10A	115.9
C2—C1—H1B	124.6	C12—C11—C16	117.45 (16)
C1—C2—C3	105.76 (14)	C12—C11—C10	123.22 (15)
C1—C2—C9	126.88 (16)	C16—C11—C10	119.26 (16)
C3—C2—C9	127.18 (14)	C13—C12—C11	120.99 (18)
C34—C3—C4	118.45 (14)	C13—C12—H12A	119.5
C34—C3—C2	107.06 (13)	C11—C12—H12A	119.5
C4—C3—C2	134.49 (14)	C12—C13—C14	120.6 (2)
C7—C4—C3	119.67 (15)	C12—C13—H13A	119.7
C7—C4—H4A	120.2	C14—C13—H13A	119.7
C3—C4—H4A	120.2	C15—C14—C13	119.32 (19)
C34—C5—C6	117.15 (14)	C15—C14—H14A	120.3
C34—C5—H5A	121.4	C13—C14—H14A	120.3
C6—C5—H5A	121.4	C16—C15—C14	120.3 (2)
C5—C6—C7	121.46 (15)	C16—C15—H15A	119.9
C5—C6—C8	119.00 (15)	C14—C15—H15A	119.9
C7—C6—C8	119.34 (15)	C15—C16—C11	121.3 (2)
C4—C7—C6	120.61 (15)	C15—C16—H16A	119.3
C4—C7—H7A	119.7	C11—C16—H16A	119.3
C6—C7—H7A	119.7	N1—C34—C5	129.96 (14)
N2—C8—C6	176.59 (18)	N1—C34—C3	107.43 (14)
C10—C9—C2	125.35 (16)	C5—C34—C3	122.59 (14)
			
C34—N1—C1—C2	0.90 (19)	C9—C10—C11—C16	169.56 (17)
N1—C1—C2—C3	−0.13 (18)	C16—C11—C12—C13	−0.4 (2)
N1—C1—C2—C9	−175.44 (15)	C10—C11—C12—C13	−177.41 (16)
C1—C2—C3—C34	−0.66 (16)	C11—C12—C13—C14	0.2 (3)
C9—C2—C3—C34	174.63 (14)	C12—C13—C14—C15	−0.1 (3)
C1—C2—C3—C4	179.82 (17)	C13—C14—C15—C16	0.3 (3)
C9—C2—C3—C4	−4.9 (3)	C14—C15—C16—C11	−0.6 (3)
C34—C3—C4—C7	−3.0 (2)	C12—C11—C16—C15	0.6 (3)
C2—C3—C4—C7	176.51 (15)	C10—C11—C16—C15	177.75 (18)
C34—C5—C6—C7	−1.5 (2)	C1—N1—C34—C5	177.01 (15)
C34—C5—C6—C8	173.32 (13)	C1—N1—C34—C3	−1.29 (17)
C3—C4—C7—C6	1.4 (2)	C6—C5—C34—N1	−178.20 (15)
C5—C6—C7—C4	0.9 (2)	C6—C5—C34—C3	−0.1 (2)
C8—C6—C7—C4	−173.94 (15)	C4—C3—C34—N1	−179.20 (13)
C1—C2—C9—C10	−8.2 (3)	C2—C3—C34—N1	1.20 (16)
C3—C2—C9—C10	177.49 (15)	C4—C3—C34—C5	2.3 (2)
C2—C9—C10—C11	173.21 (15)	C2—C3—C34—C5	−177.26 (13)
C9—C10—C11—C12	−13.5 (3)		

### Hydrogen-bond geometry (Å, °)

**Table:**

*D*—H···*A*	*D*—H	H···*A*	*D*···*A*	*D*—H···*A*
N1—H1A···N2^i^	0.90	2.19	3.043 (3)	158
C5—H5A···N2^ii^	0.93	2.66	3.416 (4)	138

Symmetry codes: (i) *x*−1/2, −*y*+3/2, −*z*; (ii) −*x*, −*y*+2, −*z*.

## References

[bb1] Brandenburg, K. & Putz, H. (2005). *DIAMOND* Crystal Impact GbR, Bonn, Germany.

[bb2] Kunzer, A. R. & Wendt, M. D. (2011). *Tetrahedron*, **52**, 1815–1818.

[bb3] Rigaku. (2005). *CrystalClear* Rigaku Corporation, Tokyo, Japan.

[bb4] Sheldrick, G. M. (2008). *Acta Cryst.* A**64**, 112–122. 10.1107/S0108767307043930 18156677

